# Medical education in Myanmar: a critical commentary

**DOI:** 10.5116/ijme.596f.9fba

**Published:** 2017-07-28

**Authors:** Myo Min Soe

**Affiliations:** 1 Medical Education Centre, School of Medicine, The University of Nottingham, UK

**Keywords:** Knowledge assessments, multiple choice question, rote learning, didactic

##  

### To the Editor

To the best of my knowledge, there has not yet been any evidence on medical education in Myanmar. This led to the academia in Myanmar having to cope with the lack of cooperation and help from the global academic community in addition to the chronic scarcity of resources, expertise and political commitment domestically. The purpose of this letter is to present a brief overview of medical education in Myanmar and to call for more help and cooperation from the global academic community.

Myanmar has a population of 52million according to the 2014 census, ranking as the 11th most populated in Asia. It has 0.568 doctors per 1000 population according to the latest WHO statistics. The average is 0.72 per 1000 in Southeast Asia. There are five civilian medical schools and one military medical school. The legacy of the military junta is visible in the fact that there is a dedicated medical school for the army less than half a million strong while there is only one medical school per approximately ten million civilians. The tuition fee is 9600kyats (£5.6) per year. There are approximately 1500 students per school. Each school accepts approximately 300 top scorers in the high school final exams as new students annually. The undergraduate course for the M.B., B.S. degree lasts five years followed by one year as a House Surgeon (the equivalent of Foundation Year One in the UK). There is no graduate entry course. In the past five years, Myanmar’s democratic reform has brought more government funding to the medical schools. The physical infrastructure is being upgraded, but the curriculum, teaching and assessments have not yet seen any significant reform. All the civilian schools use the same curriculum that has remained virtually unchanged for decades. Basic sciences are taught in the early years, and clinical training starts in year three. The Burmese language is used for speaking and English for writing.

Teaching in undergraduate medicine is mainly didactic, and educators commonly encourage rote learning. Problem-based learning is currently being piloted under the name of the integrated curriculum. Unique to Myanmar is there are private small group lectures in the evenings and weekends. Some of the teachers who work at the medical school and junior doctors who had high grades as undergraduates deliver these lectures. There has not been any research on this phenomenon but based on my experience, a vast majority of undergraduates attend private lectures. Private tuition fee per month is approximately 10000kyats (£5.7) for each subject. Students seem to feel private lectures are more convenient and easier to follow than the lectures at the medical school delivered by the same teachers. The likely reasons are the low government salary and the lack of amenities such as air conditioning in medical schools. Students also seem to rely on study notes written by teachers and high performing students in previous years. These “expert written” study notes are concise, focusing on the important topics for the exams. For students who should learn English as a second language, these notes are easier to read than the text books. Also, text books are relatively expensive, and there are not enough of them stocked in the library for all students.

Knowledge assessments use Multiple Choice Questionnaires (MCQs) and essays. Each “multiple choice” item is five True/False items sharing a common stem in which three of the options are correct, and two are incorrect. Maximum possible mark for each item is five, and the minimum is zero. Negative marking, abandoned by the GMC in the UK nearly ten years ago, is still being used to discourage guessing. One mark is awarded for every correct choice and one penalised for every incorrect choice. Single Best Answer MCQs are not used. Extended Matching Questions are included in some exams. Essays are marked against standard criteria. Skill assessments use Objective Structural Clinical Exams (OSCEs). All schools share a common question bank. The summative assessments are delivered at the same time across all schools using the same questionnaire. External examiners do not only observe but also assess the candidates. The lack of expertise in medical education is apparent in the standard setting process. The assessment boards consider neither absolute standard setting methods such as Angoff and Ebel nor post-examination psychometric analysis.  The pass mark is set at 50% for all assessments. After the exam, the pass mark is moderated if the assessment board feels the false negative (competent students who should pass but failed) rate is too high. The process is based totally on the subjective interpretation of the results by the assessment board. This type of posthoc standard setting is neither robust, transparent, consistent nor based on psychometric evidence.^1^

For postgraduates, there are clinical training courses for a diverse array of specialities. The application process is highly competitive. Only the government employees are allowed to apply. It is meant to attract more doctors to work for the government. While these clinical training courses are the equivalents of Core Training and Specialist Training in the UK, they award MSc and PhD degrees. The trainees are required to do a minor research project and submit a thesis in addition to completing clinical training. The research is often severely limited in scope and depth be cause it is not the main purpose of the course and there is little research funding, infrastructure and expertise.

To address these challenges, an independent medical education research infrastructure needs to be established. It should be used to improve the capacity of the educators and to find acceptable solutions to local issues such as private lectures. The current undergraduate question bank and the standard setting process must be overhauled thoroughly. Postgraduate clinical training courses should remove the rudimentary research component. In addition to adequate funding, more cooperation with the international scholars in medical education is vital to boost the expertise of the fledgeling academic community in Myanmar.

### Conflict of Interest

The author declares that they have no conflicts of interest.
